# Unravelling therapeutic resistance: role of cancer stem cell and microRNA in metastatic prostate cancer

**DOI:** 10.3389/fmolb.2026.1904473

**Published:** 2026-09-15

**Authors:** Hitakshi Moondra, Gautam Ram Choudhary, Dinesh Kumar Ahirwar, Mithu Banerjee, Surendra Kumar Shukla, Kamla Kant Shukla

**Affiliations:** 1 Department of Biochemistry, All India Institute of Medical Sciences, Jodhpur, Rajasthan, India; 2 Department of Urology, All India Institute of Medical Sciences, Jodhpur, Rajasthan, India; 3 Department of Biosciences and Bioengineering, Indian Institute of Technology, Jodhpur, Rajasthan, India; 4 Department of Oncology Science, University of Oklahoma Health Sciences Centre, Oklahoma City, OK, United States

**Keywords:** cancer stem cells, cluster of differentiation, drug resistance, MicroRNAs, prostate cancer, therapy resistance

## Abstract

Metastatic prostate cancer (PCa) is one of the leading causes of cancer-related deaths in men globally. This is largely attributable to the occurrence of therapeutic resistance that reduces the efficacy of the traditional treatment, such as Androgen deprivation therapy, chemotherapy, and radiotherapy. Accumulating evidence suggests that prostate cancer stem cells a rare population of Cancer Stem Cells drive therapy resistance by sustaining self-renewal, tumor plasticity, epithelial-mesenchymal transition, and metastasis. Concurrently, miRNAs are critical post-transcriptional regulators of gene expression, modulating key signalling pathways that control CSC stemness, reshaping tumor microenvironment, and conferring resistance to therapy. Dysregulation of miRNAs has been closely associated with the regulation of CSCs and the progression of metastatic PCa, and causes disease recurrence. In accordance with their targets, miRNAs can function either as oncogenic drivers that promote cancer progression or as tumor suppressors that inhibit malignant transformation and growth. Moreover, it contributes to the capacity of CSCs to withstand therapy and initiate disease recurrence. Recent advances in precision oncology have increased the pace of novel therapeutic modalities, such as CRISPR/Cas9, nanotechnology-based delivery vehicles, chimeric antigen receptor -T cell therapy, and epigenetic reprogramming strategies to counteract aberrant transcriptional states that sustain PCSC plasticity. The integration of these approaches offers a promising strategy to overcome refractoriness to treatment and improve clinical outcomes in advanced PCa. This review highlights the complex interplay between miRNAs and PCSCs in therapy resistance of metastatic PCa, and explores their translational potential in shaping durable and more effective clinical interventions.

## Graphical Abstract

**Figure:**
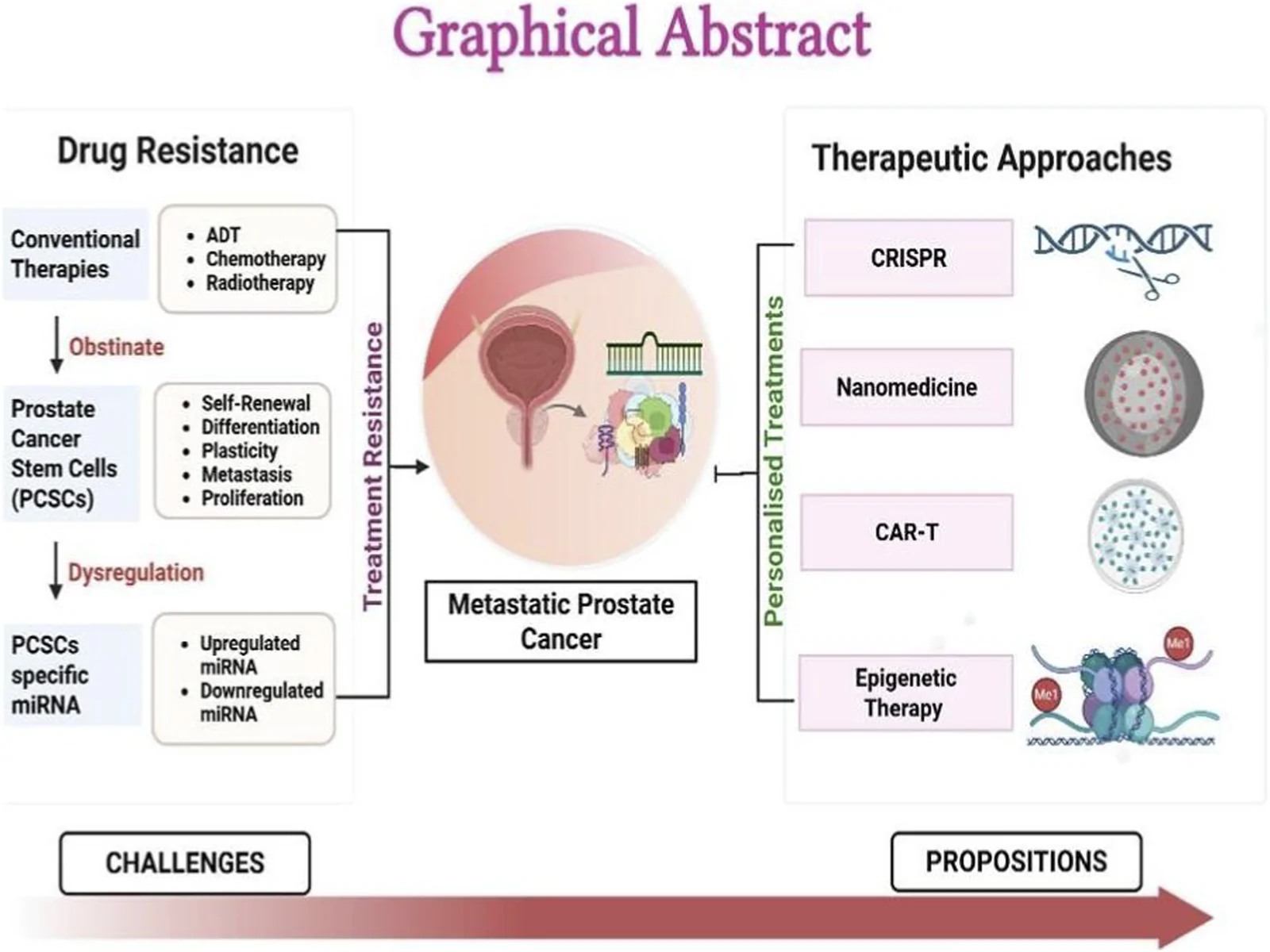

## Introduction

Prostate cancer (PCa) represents one of the major health issues, the second most frequently diagnosed, and the fifth leading cause of cancer-related deaths in men worldwide (Kania et al., 2025; Rawla, 2019; Wang L. et al., 2022). According to GLOBOCAN 2020, there were approximately 1,414,259 newly diagnosed cases and 375,304 related deaths (James et al., 2024). It is projected that the prevalence of PCa in the world will rise; by the year 2040, there will be about 2.4 million cases and approximately 712,000 deaths, mainly driven by population growth and ageing of the population (Schafer et al., 2025; Shoag and Barbieri, 2016). PCa is a heterogeneous disease with variable clinical outcomes in patients (Chen et al., 2023; Huff et al., 2006). Although the treatment options have improved considerably, late PCa has been identified to have a difficult prognosis, and most men succumb to the disease (Berenguer et al., 2023; Li et al., 2025). It is a cancer that is dependent on androgens; therefore, the androgen receptor (AR) plays a crucial role in disease progression (Li et al., 2025; Choi et al., 2022). Androgen deprivation therapy (ADT) is a standard treatment for advanced PCa, which includes either surgical or chemical castration to reduce testicular androgens and tumor progression (Crawford et al., 2019; Murayama and Gotoh, 2019). Conventional treatments often lead to resistance and poor therapeutic response, highlighting the urgent need for innovative strategies to combat this formidable foe (Li et al., 2025; Ehsani et al., 2021). Even with improved treatment options, CSCs that are refractory to treatment have emerged, and it has been nearly impossible to cure the metastatic PCa (Murayama and Gotoh, 2019; Liang et al., n.d.; Safa, 2020). The latter cells are characterized by their capacity to self-renew, acquire a heterogeneous tumor cell population, and become resistant to standard therapies (El-Tanani et al., 2025; Chu et al., 2024). CSCs have been linked to disease progression, recurrence, metastasis, ADT resistance, and chemotherapy in PCa (Tu and Lin, 2012; Romańska-Knight and Abel, 2011). The identification and functional characterization of prostate cancer stem cells (PCSCs) have highlighted their role in driving epithelial-mesenchymal transition (EMT), immune evasion, and plasticity, offering promising avenues for improving therapeutic outcomes in metastatic PCa (Liu and Tang, 2011).

MicroRNAs (miRNAs) have proven to be key players in the regulation of CSC biology, among other molecular regulators (Khan et al., 2019; Mao et al., 2025). They are small non-coding RNA molecules that are post-transcriptionally involved in the regulation of gene expression and are involved in cell growth, cell death, metastasis, and differentiation (Mao et al., 2025; Fang et al., 2015). PCa dysregulation of certain miRNAs has been implicated to influence CSC homeostasis, tumor microenvironmental interactions, progression, and therapeutic resistance (Li W.(Jess) et al., 2022). Despite the immense therapeutic potential of targeting miRNAs, several hurdles remain to be resolved in the development of CSC-specific mechanisms (Coradduzza et al., 2022).

In the novel therapeutic avenues, CRISPR/Cas9-based genome editing is a platform to precisely silence oncogenic or tumor suppressive miRNA. It regulates the maintenance of PCSCs, and thereby directly target the molecular drivers of stemness and metastatic progression (Hazafa et al., 2020). Simultaneously, the CAR-T cell-mediated immunotherapy has been engineered to recognize CSC-specific surface markers, including CD44, EpCAM, and CD133. In addition to miRNA modulation, this combination increases tumor recognition as well as reverses immunosuppression that shields PCSCs (Lee et al., 2025). Delivery systems based on nanomedicine result in encapsulation and specific delivery of miRNA mimics, miRNA-inhibitors, or miRNA-combinations. These formulations ensure selective accumulation in tumor tissues and reduced systemic toxicity, thus enhancing efficacy against PCSC therapy-resistant (Arabi et al., 2016). Moreover, epigenetic therapies reestablish distorted transcriptional networks and miRNA expression, and thus, affect PCSC plasticity and maintenance. Targeted miRNA therapeutics in combination with conventional therapies lead to the restoration of tumor-suppressive pathways and overcome conventional therapeutic resistance (Majid et al., 2013).

Together, these new approaches highlight the key role of the dysregulated miRNAs in PCSCs and their role in precision medicine. Thus, ranging from genome editing to nanomedicine, immunotherapy, and epigenetic reprogramming, these advancements aim to improve PCa management and increase the outcome of patients. The integration of miRNA biology, CSC targeting, and therapeutic technology, therefore, represents a promising and cutting-edge paradigm for addressing aggressive, metastatic, and treatment-resistant PCa. This review aims to provide a comprehensive overview of recent advances made in PCSC dynamics, regulatory activities of miRNAs in CSC-related pathways, as well as the development of PCSC-specific miRNA-targeted therapies.

## Literature search strategy

This article is a narrative review of the existing literature and does not follow a systematic review methodology; therefore, formal inclusion/exclusion criteria and PRISMA reporting guidelines are not applicable. Relevant literature was identified through a comprehensive search of three major electronic databases: PubMed/MEDLINE, Scopus, and Google Scholar. The following search terms and their combinations were used: ‘prostate cancer” “prostate cancer stem cells”, “cancer stem cells prostate”, “PCSC”, “miRNA prostate cancer”, “microRNA therapeutic resistance”, “metastatic prostate cancer”, “castration-resistant prostate cancer”, “CRPC stem cells”, “Wnt Notch Hedgehog prostate cancer”, “miRNA cancer stem cell regulation”, “CRISPR prostate cancer”, “nanomedicine miRNA delivery”, “CAR-T prostate cancer”, and “epigenetic therapy prostate cancer”. The search was not restricted by study design and encompassed peer-reviewed original research articles, review articles, meta-analyses, and clinical trial reports published in the English language up to December 2025. Additional references were identified through manual screening of the bibliographies of included articles. Studies addressing the molecular biology of PCSCs, miRNA-mediated regulation of stemness and drug resistance, tumour microenvironment interactions, and emerging therapeutic strategies targeting PCSCs were considered relevant and included in this review. It is important to note that this review is not systematic or exhaustive; rather, article selection prioritized studies of mechanistic and translational relevance to CSCs, miRNA regulation, metastatic progression, and therapy resistance in PCa.

## Human prostate epithelium cellular hierarchy

The human prostate epithelium exhibits a well-defined cellular hierarchy that resembles glandular tissues and serves as a foundation for prostate development, homeostasis, and tumorigenesis. The prostate gland is lined by pseudostratified epithelium that consists of three different cell types, including basal cells, luminal cells, and neuroendocrine cells. Basal prostate stem cells express a set of surface and intracellular markers, including cytokeratin 5 (CK5), CK14, p63, integrin 21, CD133, and CD44 (Zhang D. et al., 2018; Wilson et al., 2017; Kasper, 2008). These cells tend to be androgen-independent and retain their regenerative ability even in the androgen-deprived conditions (Banerjee et al., 2023). On the contrary, luminal epithelial cells are the most common cell type in the prostate, and the cells are characterized by the expression of CK8 and CK18, an increase of AR in the cells, and cell surface proteins such as CD24 and CD26, as well as secretory proteins like prostate-specific antigen (PSA). These cells are dependent on androgen and show an elevated response to ADT (Verma et al., 2023). Additionally, an “intermediate” cell population has been discussed, characterized by basal localization and its co-expression with basal and luminal markers. However, the transition state and its unique lineage in these cells remain unclear (Wang Z. A. et al., 2013). Neuroendocrine cells, which are characterized by markers such as chromogranin A, Enolase 2 (NSE), and synaptophysin, are present within the prostate, although their precise function in PCa remains unknown (Zazzo et al., 2015). Figure 1 illustrates the cellular hierarchy of human prostate epithelium that was organised into a well-defined lineage comprising basal, luminal, and rare populations of neuroendocrine cells sustained by a group of progenitor or stem-like cells. Notably, this hierarchy offers a basis of cellular plasticity and tumor heterogeneity, as PCSCs are believed to originate from either basal or luminal progenitor compartments, depending on oncogenic drivers and microenvironmental cues.

**FIGURE 1:**
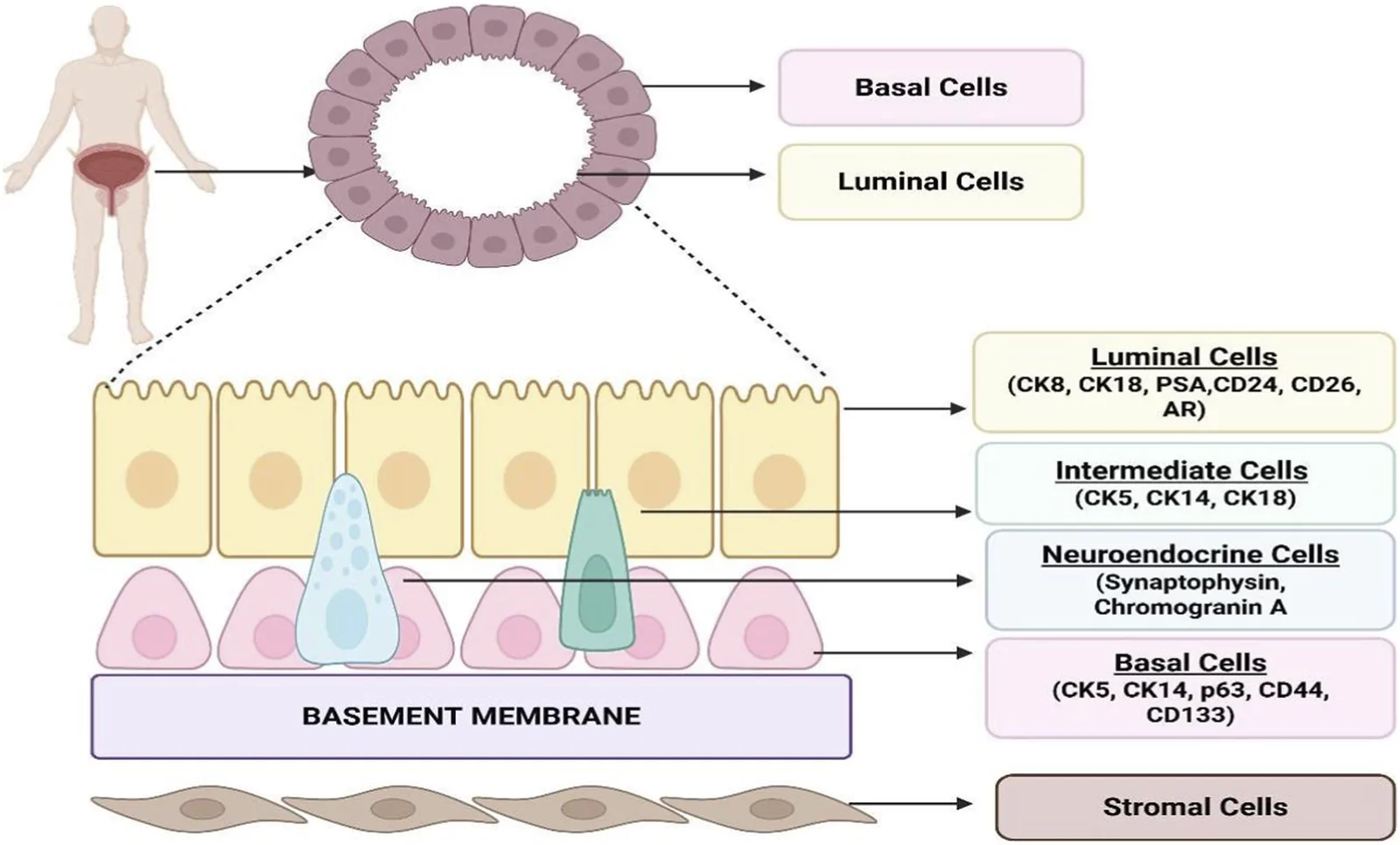
Cellular hierarchy of the human prostate epithelium. The prostate epithelium comprises luminal cells (CK8, CK18, PSA, CD24, CD26, AR), basal cells (CK5, CK14, p63, CD44, CD133), intermediate cells (CK5, CK14, CK18), and neuroendocrine cells (synaptophysin, chromogranin A), supported by the basement membrane and stromal cells. Aberrant transformation within these epithelial compartments, particularly basal and luminal cells, is proposed to give rise to prostate cancer stem cells (PCSCs). This figure was created using BioRender and accessed on 30 October 2025.

## Limitations of PCSC isolation and characterization approaches

Despite considerable progress in identifying and enriching PCSCs, current isolation strategies carry inherent limitations that warrant careful consideration. Surface marker-based sorting approaches employing CD44, CD24, CD133, and α2β1 integrin, or combinations thereof, have demarcated a discrete stem-like population using Fluorescence Activated Cell Sorting (FACS) or Magnetic-Activated Cell Sorting (MACS) (Liao et al., 2016). However, no single marker is uniquely specific to PCSCs, and their expression is highly dynamic, varying with tumor stage, EMT plasticity, and the surrounding tumor microenvironment. Furthermore, the combinatorial marker profiles used to define PCSCs vary considerably across studies, complicating cross-study comparisons and limiting reproducibility. Similarly, sphere-forming assays are widely used as a surrogate functional readout for self-renewal and stemness, but are influenced by culture conditions such as seeding density, growth factor composition, and introducing inter-laboratory variability (Pastrana et al., 2011). Furthermore, *in vivo* xenograft transplantation assays are considered the functional gold standard for validating tumor-initiating capacity. These are constrained by their reliance on immunocompromised host models that do not recapitulate the native tumor immune microenvironment, and by the substantial time and cost (He et al., 2023). Additionally, a major translational limitation arises from the predominant use of established PCa cell lines, including DU-145, PC-3, and LNCaP, which only partially reflect the genetic, epigenetic, and phenotypic heterogeneity of primary or metastatic patient-derived tumors (Namekawa et al., 2019). Collectively, these methodological constraints underscore the need for complementary validation approaches, including patient-derived organoids and single-cell transcriptomic profiling, to more accurately define and target the PCSC compartment in future therapeutic development.

## Prostate cancer: molecular mechanism of drug resistance

Drug resistance is the major obstacle in PCa treatment, which often leads to therapeutic failure, poor patient survival, disease recurrence, and higher mortality rates (Chen et al., 2025). Despite significant advances in therapeutic approaches, the metastasis to distant sites often occurs, leading to a treatment-refractory stage (Wade and Kyprianou, 2018; Nakazawa et al., 2017). The loss of efficacy in conventional treatment is attributed to the evolution of multifactorial resistance mechanisms, including diminished intracellular accumulation of drugs, impaired apoptosis, enhanced DNA damage, and tumor microenvironment remodelling (Wade and Kyprianou, 2018; Galletti et al., 2017; Nikolaou et al., 2018; Damia and Garattini, 2014). Among the cornerstone treatments for castration-resistant prostate cancer (CRPC), the most aggressive and lethal form of the disease, are the AR-targeted therapies such as enzalutamide and abiraterone, and the chemotherapeutic agent docetaxel, and each of these therapies acts on the AR pathway either directly or indirectly (Thakur et al., 2018). Docetaxel is the first-line standard treatment, and usually it enhances survival through induction of mitotic arrest and apoptosis (Magadoux et al., 2014). Conversely, an obstacle in the metastatic CRPC frequently leads to failure in the treatment, tumor recurrence, and disease progression (Hashemi et al., 2023). Similarly, next-generation medications like enzalutamide and abiraterone are potent AR signalling pathways inhibitors that are primarily used in the treatment of advanced metastatic CRPC (Shah et al., 2022; Magadoux et al., 2014; Rodriguez-Vida et al., 2015; Lavaud et al., 2020). Enzalutamide is an AR antagonist that acts by blocking androgen binding, inhibiting the AR nucleus translocation, and preventing AR-mediated transcriptional factor, ultimately suppressing PCa growth (Nadal and Bellmunt, 2016). The therapeutic resistance commonly arises through several AR-dependent mechanisms, including AR gene amplification, point mutation, and the constitutively active splice variants such as AR-V7, and drives transcription independent of androgen (Maylin et al., 2021; Huang et al., 2026; Chen et al., 2022; Jacob et al., 2021). On the other hand, abiraterone inhibits the enzyme CYP17A1 that is essential for androgen production in the adrenal glands, testes, and tumor tissues. This leads to marked reduction in circulating and intra-tumoral androgen levels, thereby depriving cancerous cells of the ligands required for AR activation (Abiraterone acetate in the treatment of prostate cancer, 2018). Clinical efficacy of both drugs is high in deceleration of the disease progression and prolonged survival in patients with metastatic CRPC (Yanagisawa et al., 2025; Swami et al., 2020; Litwin and Tan, 2017). The primary reason for the emergence of drug resistance in advanced PCa is the survival and expansion of these therapy-resistant PCSCs. Unlike the bulk tumor cells, which are generally responsive to the conventional therapies, PCSCs possess unique biological properties that enable them to escape drug-induced cytotoxicity (A review of the efficacy of prostate cancer therapies against castration-resistant prostate cancer, 2025). They often reside in either a quiescent or slow-dividing state, making them less susceptible to drugs that target rapidly dividing cells. Additionally, PCSCs exhibit low expression of AR, rendering AR-targeted therapy agents like enzalutamide and abiraterone ineffective against this population (Qin et al., 2020). Nonetheless, the mechanisms of resistance are multifaceted, and PCSC, along with the associated intra-tumoral plasticity, play a pivotal role in sustaining tumor growth, mediating therapeutic resistance, and driving disease progression. Table 1 below gives an overview of the molecular mechanisms involved in resistance to various therapeutic agents applied in the management of CRPC and their effects on tumor progression, metastatic spread, and resistance to treatment.

**TABLE 1:** Different therapeutic agents utilized in CRPC management.

Drug name	Target	Description	Key findings	References
Docetaxel	Microtubules	A taxane-based chemotherapeutic agent functions by stabilizing microtubules, disrupting mitotic processes, and triggering apoptosis in proliferating cells. AR-V7 exhibits reduced susceptibility to microtubule stabilization by taxanes, thereby contributing to therapeutic resistance.	Docetaxel is the standard first-line therapy for metastatic CRPC, but resistance arises through mechanisms such as drug efflux (ABC transporters), EMT, and accumulation of CD44+/CD133+ PCSCs with low mitotic activity. Altered expression of class IV β-tubulin and mutations in class I β-tubulin further impair microtubule polymerization, driving resistance.	Chen et al. (2025), Qin et al. (2020)
Cabazitaxel	Microtubules	Cabazitaxel, a second-generation taxane, remains effective in docetaxel-resistant PCa by maintaining activity even in tumors with high P-glycoprotein expression. Resistance to cabazitaxel can emerge through overexpression of the ETS-related gene (ERG) in prostate cells.	It enhances survival in patients with docetaxel-resistant disease by more effectively targeting quiescent and therapy-resistant CSCs compared to docetaxel. Disrupting ERG interactions can help restore treatment sensitivity.	Nimmakayala et al. (2019), Yadav and Desai (2019), Collins et al. (2005b)
Enzalutamide	AR	A strong inhibitor of AR signalling that prevents AR nuclear entry, DNA binding, and recruitment of transcriptional coactivators.	It improves survival in metastatic CRPC, but resistance commonly arises through AR splice variants such as AR-V7, enhanced intratumoral androgen production, or stem-like cancer cell populations that bypass AR dependence.	Shah et al. (2022), Collins et al. (2005a), Unno et al. (2021)
Abiraterone	CYP17A1 enzyme	It blocks CYP17A1, a critical enzyme in androgen biosynthesis, thereby lowering both systemic and intra-tumoral androgen levels. Resistance develops when PCa reactivates androgen production despite treatment.	It markedly improves outcomes in metastatic CRPC. Resistance emerges through mechanisms such as glucocorticoid receptor activation and lineage plasticity driven by PCSCs. Reactivation of androgen biosynthesis contributes to abiraterone resistance in patients.	Jacob et al. (2021), Liu (1994)

## Origin of cancer stem cells (CSCs) in prostate cancer

The concept of CSCs was first introduced in the hematologic malignancies during the late 1990s and was later extended to various solid tumors, including PCa (Chu et al., 2024; Nimmakayala et al., 2019). These were characterized as a rare subpopulation of tumor cells endowed with the ability to self-renew, differentiate into multiple cell types, and initiate tumor formation (Qin et al., 2020). In the context of PCa, there are symmetrical and asymmetrical divisions, which lead to the development of tumor heterogeneity (Romańska-Knight and Abel, 2011). This enables them to maintain the stem cell pool, generate cell lineages, complexity, progression, and therapeutic resistance (Yadav and Desai, 2019). A pivotal advancement in this field was made in 2005, when Collins and colleagues successfully isolated PCSCs from human prostate tumors (Collins C. A. et al., 2005). Their findings revealed that these cells possess robust self-renewal capabilities and were able to differentiate into a non-clonal heterogeneous population (Collins C. A. et al., 2005). This landmark discovery highlighted the role of PCSCs in initiating tumors and sustaining cellular diversity within the tumor microenvironment (Collins A. T. et al., 2005). Although substantial progress has been made, the precise cellular origin of PCSCs remains a matter of debate (Liang et al., n.d.).

Based on earlier research, tumor-initiating cells in PCa could be of either the basal or luminal compartments of prostate epithelium (Banerjee et al., 2023). Markedly, Zhang et al*.* emphasized the crucial role of luminal progenitor cells in stimulating PCa onset and advancement (Zhang D. et al., 2018). PCSCs have been identified to be the key regulators of tumor occurrence, growth, and metastasis (Banerjee et al., 2023). Two types of luminal progenitor cells and basal stem/progenitor cells that exist in the normal prostate epithelium are potential sources of PCSCs (Verma et al., 2023). The lineage tracing studies conducted by Wang et al*.* uncovered that PCSCs are located in the basal and luminal regions of the prostate epithelium (Wang Z. A. et al., 2013). Even though tumors that occur as a result of luminal progenitors are typically linked with the most common subtype of PCa adenocarcinoma. Although basal cell-based tumors are associated with more aggressive types, such as neuroendocrine PCa (Unno et al., 2021). Collectively, these findings underscore the complexity of the biology of PCSCs and emphasize a more in-depth study of their origin and functional heterogeneity in disease development and therapeutic resistance.

## Molecular markers of prostate cancer stem cells

The identification and characterization of PCSCs depend on the specific surface markers’ expression that distinguishes them from the bulk tumor population. These biomarkers provide mechanistic insights into their roles in cancer initiation, expansion, dissemination, and therapeutic escape (Romańska-Knight and Abel, 2011; Tu and Lin, 2012). Among these, CD44 is one of the most widely recognized and functionally validated markers. It is a single-pass type I transmembrane glycoprotein involved in cell adhesion and migration, acting as a receptor component of the extracellular matrix, such as hyaluronic acid (Liu, 1994). In PCa, CD44^+^ cell populations have been consistently shown to exhibit hallmark features of cancer stem-like cells (Ajani et al., 2015; Zhang et al., 2016; Wang L. et al., 2013). These include enhanced clonogenicity, spheroid formation, and significantly increased tumor-initiating potential in both *in vitro* and *in vivo* models (Tsao et al., 2019; Harris and Kerr, 2017). Functionally, CD44^+^ PCa cells tend to be quiescent and more often resistant to conventional therapies such as chemotherapy and radiotherapy (Li et al., 2021). Clinically, the presence of CD44^+^ PCSCs has been linked to higher Gleason scores, advanced tumor stage, and poorer overall survival in patients with metastatic PCa (Binal et al., 2020; Castellón et al., 2012). Similarly, a study by Shukla et al*.* demonstrated a significant increase in PCSC markers, particularly CD44^+^/CD24^-^ and CD44^+^/CD133^+^ subpopulations. These CD44^+^ cells contribute to disease aggressiveness in localized and metastatic PCa compared to benign prostatic hyperplasia (Shukla et al., 2023).

CD24 is a glycosylphosphatidylinositol (GPI)-anchored cell surface protein that has gained recognition as a key marker and functional regulator of PCSCs, significantly influencing tumorigenesis and progression as well as treatment resistance (Hermann et al., 2010). Whereas the normal prostate epithelial cells usually express low or none of CD24, it is significantly upregulated in high-grade prostatic intraepithelial neoplasia (PIN), primary prostate tumors, CRPC, and metastatic lesions (Kristiansen et al., 2004; Hurt et al., 2008; Aigner et al., 1998; Jaggupilli and Elkord, 2012). This observation is supported by the Cancer Genome Atlas (TCGA) database, which revealed that the expression of CD24 mRNA correlates positively with advanced stages of PCa and metastasis to lymph nodes (Weng et al., 2019). Functionally, the CD24^+^ PCSCs exhibit hallmark stemness features, such as enhanced self-renewal capacity, increased sphere-forming capacity, elevated tumorigenicity in xenograft models, and sustained proliferative ability over time (Deshmukh et al., 2017). Therefore, it contributes to cancer resistance against classical therapies, including radiotherapy and chemotherapeutic drugs, such as doxorubicin and cisplatin, and targeted therapies (Tang, 2022; Yang et al., 2023). Supporting this, David et al*.* proved that CD24^+^ mesenchymal stem/progenitor cells form spheres *in vitro,* and they replicate the local invasion and distant metastasis *in vivo* (Mulholland et al., 2012). Although compelling experimental evidence has demonstrated the multifaceted role of CD24 in the progression of PCSCs, its clinical significance has remained underexplored, highlighting the need for further investigation to determine its potential role as a prognostic biomarker and therapeutic agent.

Another widely recognized marker is CD133 (Prominin-1), a well-characterized transmembrane glycoprotein initially identified as a marker of primitive hematopoietic and neural stem cells, which has gained considerable attention as a key marker for PCSCs (Kanwal et al., 2018). Notably, within the prostate, a subset of basal epithelial cells expressing high concentrations of α2β1 integrin also expresses CD133. Such cells have a high proliferative capacity and the ability to regenerate a completely differentiated epithelium, including prostatic secretory structures (Trerotola et al., 2010). Functional analyses have shown that CD133+ cells isolated from both PCa tissues or cell lines exhibit enhanced clonogenicity, robust sphere-forming ability, and long-term proliferation, key characteristic features of cancer stemness (Ayla et al., 2024; Koukourakis et al., 2023). These cells have enhanced anchorage-independent growth, enhanced proliferation, and clonogenic efficiency as opposed to their CD133 counterparts (Ayla et al., 2024). The high-grade tumors and metastatic lesions are enriched with CD133+ cells whose frequency significantly increases in CRPC, which highlights the clinical relevance of these cells (Yang et al., 2022). The existence of CD133, which has a positive expression in a clinical setting, has been linked to poor prognostic factors such as a higher Gleason score, lower survival, and an increased risk of recurrence and metastasis (Yang et al., 2022). In line with this, research by Rajnee and colleagues has identified CD133+ circulating tumor cells (CTCs) in advanced CRPC patients, before and after therapeutic intervention (Kanwal et al., 2018). It is worth noting that targeting CD133 expression was confined to a distinct subset of CTCs, indicative of a more aggressive and therapy-resistant phenotype (Reyes et al., 2015; Armstrong et al., 2011; Pal et al., 2015). Therefore, targeting this approach to CD133^+^ PCSCs represents a critical strategy in the development of more effective and long-lasting treatment aimed at eliminating the tumor-forming centre of PCa.

## Regulation of stem cell population by oncogenic signalling pathways

Although the precise molecular mechanisms underlying the morphogenetic processes in PCa remain unclear, it is well established that AR signalling, along with key oncogenic pathways such as RAS-RAF, PTEN-PI3K-AKT, Wnt, Notch, and Hedgehog, plays a central role in driving PCa initiation and progression (Kushwaha et al., 2022; Verma et al., 2020; Gu et al., 2007; Federer-Gsponer et al., 2020; Katoh, 2017).

### Wnt signalling

Emerging evidence indicates that the Wnt signalling pathway, through both direct and indirect mechanisms, contributes to the malignant transformation of prostate epithelial cells, the development of primary PCa, and its progression to CRPC (Zhang Z. et al., 2018; Armenia et al., 2018). The canonical Wnt signalling pathway is activated when Wnt ligands bind to Frizzled receptors and LRP5/6 co-receptors, resulting in the inhibition of the β-catenin destruction complex. This leads to the stabilization of nuclear translocation of β-catenin, which interacts with TCF/LEF transcription factors to activate Wnt target gene expression (Zhang K. et al., 2017). In PCa, the AR functions as an interacting partner of β-catenin, and both are frequently expressed in tumor tissues. Their elevated expression levels are positively correlated with higher Gleason grades, increased metastatic potential, enhanced tumor aggressiveness, and elevated serum PSA levels (Velho et al., 2020). Zhang et al*.* have demonstrated that the nuclear receptor NURR1 plays a pivotal role in promoting both EMT and cancer stemness in PCa by directly regulating the transcription of CTNNB1 and activating β-catenin signalling (Zhang et al., 2024). Persistent aberrant activation of canonical Wnt signalling in PCa is often mediated by enhanced recruitment of β-catenin and AR to regulatory elements of oncogenes such as c-MYC, thereby upregulating Myc and its downstream targets, including CD44, OCT4, SOX2, and TGF-β. This signalling cascade subsequently induces the expression of multiple oncogenes and stemness-related factors such as MMP7, MET, Cyclin D1, SIX2, CD44, CD133, ALDH1A1, SOX2, and NANOG, which collectively maintain PCSC properties and promote tumor progression (Liu et al., 2022; Bisson and Prowse, 2009; Lee et al., 2016; Bhattacharyya et al., 2017; Li et al., 2017). In addition to this, Patrawala et al*.* demonstrated that CD44^+^ PCa cells exhibit elevated expression of OCT-3/4, BMI-1, and β-catenin, further validating their stem-like phenotype (Patrawala et al., 2006). Bisson et al*.* reported that Wnt3a treatment significantly increased prostasphere formation and self-renewal capacity, accompanied by elevated nuclear β-catenin levels and upregulation of epithelial and stemness markers such as keratin 18, CD133, and CD44 (Bisson and Prowse, 2009). Zhu et al*.* emphasized the anti-cancer role of capsaicin against PCSCs, showing that it significantly inhibits GSK3β phosphorylation, reduces β-catenin levels along with its downstream targets c-MYC and Cyclin D1, and blocks the nuclear translocation of β-catenin (Zhu et al., 2020). Although Wnt signalling is also regulated by paracrine interactions, the loss of SFRP1 expression in epithelial cells contributes to the activation of stem-like properties in PCa. The upregulation of pluripotency markers such as SOX2, OCT4, and NANOG, which are closely associated with cancer stem cell-driven tumor progression and resistance to therapies such as bicalutamide (Losada-García et al., 2023). For instance, miR-1301-3p is frequently upregulated in PCSCs and promotes Wnt signalling by directly targeting and suppressing key inhibitory regulators such as GSK3β and SFRP1 through binding to their 3′untranslated regions. This suppression enhances β-catenin stabilization and drives the transcriptional activation of downstream stemness-associated genes, including OCT4, SOX2, NANOG, CD44, KLF4, c-MYC, and MMP2 (Song et al., 2018). Furthermore, aberrant AR activation has been shown to trigger the insulin-like growth factor 1 (IGF1) signalling cascade in prostatic basal epithelial cells, facilitating their oncogenic transformation. This, in turn, augments Wnt/β-catenin signalling in transformed cells, reinforcing PCa progression (Kim et al., 2022). In this context, Pedro et al*.* investigated the effects of AR-targeted therapies, such as enzalutamide and abiraterone, and found that activation of the Wnt signalling pathway negatively influences patient outcomes, ultimately contributing to reduced overall survival (Velho et al., 2020). To address the consequences, VK Kim and colleagues demonstrated that Wnt inhibitors, including ICG-001 and iCRT3, can disrupt the interaction between AR and β-catenin, thereby suppressing AR-mediated transcriptional activity and inhibiting the proliferation of PCa cells (Kim et al., 2022; Atawia et al., 2023). Riza et al*.* reported that pre-treatment with a Wnt/β-catenin inhibitor CCT036477 (iWnt) combined with salinomycin significantly reduced PCa cell migration by downregulating vimentin and upregulating E-cadherin, thereby enhancing the effectiveness of subsequent cabazitaxel therapy (Serttas and Erdogan, 2023).

### PI3K/AKT signalling

Another signalling pathway is the phosphoinositide 3-kinase (PI3K)/AKT signalling pathway, which plays a central role in regulating critical cancer hallmarks, including cell survival, metastasis, and metabolic reprogramming (He et al., 2021). Growing evidence highlights its essential involvement in PCa progression, contributing to malignant transformation, primary tumor development, and the emergence of CRPC (Carver et al., 2011). Persistent activation of this pathway arises through various mechanisms, predominantly genomic alterations such as mutations in PIK3CA, AKT, mTOR (mechanistic target of rapamycin), or loss of the tumor suppressor gene PTEN. These changes lead to sustained oncogenic signalling and therapeutic resistance (Taylor et al., 2010; Vivanco and Sawyers, 2002). Alterations in the PI3K/AKT/mTOR axis are observed in approximately 40% of early-stage PCa cases and increase to 70%–100% in advanced disease. Notably, loss of PTEN results in constitutive PI3K activation and is detected in about 30% of primary tumors and up to 60% of CRPC cases, and dysregulation is strongly linked to ADT resistance, tumor progression, and poor clinical outcomes (El Sheikh et al., 2008; Reid et al., 2010; Taylor et al., 2010; Carver et al., 2011). For instance, Lin et al*.*, revealed that PTEN functions as a negative regulator of AR activity and that the interaction between PTEN and AR is critical for mediating PTEN’s tumor-suppressive effects, including the induction of apoptosis in PCa cells (Lin et al., 2004). Clinically, elevated levels of phosphorylated AKT have been predominantly observed in high-risk PCa specimens with higher Gleason scores, in contrast to less aggressive cases (Malik et al., 2002; Lotan et al., 2016). Additionally, the PI3K/AKT pathway is activated in DU145 PCa sphere cells, where high activity of this pathway is essential for both sphere formation and maintenance of stem-like cell populations (Rybak et al., 2011). In support of this, Rybak et al*.* showed that PTEN knockdown in DU145 cells led to an increased population of stem-like cells expressing CD133^+^/CD44^+^ markers, underscoring the role of PI3K/AKT signalling in promoting cancer stemness (Romańska-Knight and Abel, 2011). PCa cells cultured under sphere-forming conditions exhibited elevated expression of PI3K subunits p110α and p110β, along with enhanced phosphorylation of downstream effectors such as Akt1, GSK3β, FoxO1a, and FoxO3a (Dubrovska et al., 2009). This suggests that FoxO3a may act as a negative regulator of PCSCs by inhibiting their maintenance and promoting differentiation (Birkenkamp et al., 2007). Recent findings reveal that combined treatment with quercetin and midkine (MK) knockdown significantly enhances apoptosis, induces G1 phase cell cycle arrest, and suppresses the migratory capacity of both PC3 cells and CD44^+^/CD133^+^ PCSCs. These effects are associated with downregulation of the PI3K/PTEN signalling pathway, highlighting a potential combinatorial strategy for targeting PCSCs (Erdogan et al., 2018; Filippi et al., 2025). Notably, elevated expression of miR-133a-3p attenuates CSCs properties by inhibiting the PI3K/AKT signalling pathway through direct suppression of upstream regulators such as EGFR, FGFR1, IGF1R, and MET, ultimately reducing the bone metastatic potential of PCa (Hou et al., 2020). In line with these findings, Yi et al*.* reported that THBS4 (Thrombospondin-4) promotes tumor progression in PCSCs by activating the PI3K/AKT pathway, which in turn upregulates key stem cell markers including CD133^+^, CD13^+^, and OCT-4, thereby sustaining the stem-like phenotype (Hou et al., 2020). To counteract these tumor-promoting effects, treatment with the PI3K inhibitor LY294002 was found to reduce sphere formation *in vitro*, while the dual PI3K/mTOR inhibitor NVP-BEZ235 significantly decreased the CD133^+^/CD44^+^ stem-like cell population *in vivo*, ultimately delaying tumor development and progression (Dubrovska et al., 2009; Dubrovska et al., 2010).

### Notch signalling

The Notch signalling pathway is crucial in the maintenance and regulation of PCSCs, significantly contributing to oncogenesis, malignant progression, dissemination, and therapeutic refractoriness (Centonze et al., 2020; Zhang L. et al., 2017; Guo et al., 2017). Wang et al*.*, revealed that AR activity enhances the oncogenic activity of Jagged 1, the key Notch ligand, thereby promoting cell proliferation in PCa cell lines (Wang et al., 2015). Supporting this, Yu et al*.* reported that Jagged 1 overexpression in PCa stimulates tumor progression by activating the Notch-Akt signalling cascade, which is also enhanced by AR signalling (Yu et al., 2014). According to research by Wang and colleagues, reported that the tumorigenic and chemo-resistant capabilities of PCSCs are associated with the Notch signalling (Wang L. et al., 2020). It is worth noting that the elevated Notch activity has been continuously observed in the docetaxel-resistant PCSC, wherein it facilitates greater tumorigenicity (Domingo-Domenech et al., 2012; Su et al., 2024). In this regard, Oktem et al*.* reported a small subpopulation of DU145 PCSCs expressing both CD133 and CD44, and confirmed that Jagged1, Delta-like 3, and Notch-1 are highly upregulated, which implies a close correlation with tumor aggressiveness and progression in PCa (Oktem et al., 2014). However, suppressing the activity of NOTCH1 by overexpression of miR-200 and let-7 has been shown to effectively reduce sphere-forming capacity and suppress EMT-related markers in PCa cells (Cheng et al., 2021). Further insights by Cheng and colleagues suggested that direct interaction of mesenchymal stem cells (MSCs) with the tumor microenvironment improves PCa stemness by stimulating the Jagged1-Notch1 signalling pathway. Importantly, selective inhibition of this pathway suppressed markedly MSC-induced tumorigenicity *in vitro* and *in vivo* (Cheng et al., 2021). Mechanistically, Notch pathway activation depends on γ-secretase, an enzyme that triggers proteolytic cleavage of the Notch receptors, releasing the Notch intracellular domain (NICD) - a key step to induce downstream transcriptional signalling. Consequently, γ-secretase inhibitors (GSIs) have emerged as promising agents for blocking Notch signalling (Venkatesh et al., 2018; Wang L. et al., 2020). In this regard, the pharmacological Notch pathway inhibitor PF-03084014 has shown efficacy in activating the chemosensitivity of PC-3 PCSCs, promoting the effect of docetaxel-induced apoptosis and cell cycle arrest, and effectively suppressing their self-renewal capacity, both *in vitro* and *in vivo* (Wang L. et al., 2020; Cui et al., 2015; Venkatesh et al., 2018).

### Sonic Hedgehog signalling

The Sonic Hedgehog (SHH) signalling pathway plays a pivotal role in the regulation of cell renewal and cell survival by binding through its ligands - Sonic, Desert, and Indian Hedgehog - which bind to the cell membrane receptors, Patched (PTCH) and Smoothened (SMO) (Niyaz et al., 2019; Yao and Chuang, 2015). The interaction results in activation and nuclear translocation of the GLI transcription factor, driving the expression of the genes involved in cell proliferation, survival, and maintenance (Hazazi et al., 2024). The proliferation and development of PCSCs have been closely linked to aberrant SHH pathway activation (Zunich et al., 2012; Chang et al., 2011). Chen and others have shown that the HH/GLI signalling increases AR activity, hence promoting the proliferation of androgen-independent PCa cells, even in a low-androgen environment (Chen M. et al., 2010; Chen et al., 2009). Additionally, GLI1 can be a promising PCSCs marker with a major role in maintaining stemness and cancer growth (Wang et al., 2023). Lili et al*.* reported that there is a positive correlation between GLI1 and the stemness marker, CD44, and survival analysis indicated that high expressions of both markers correlated with poor clinical outcomes and poor survival in PCa patients (Lv et al., 2018). In addition, SHH pathway activation in CD133^high^/CD44^high^ PCSCs has been implicated in promoting resistance to zoledronic acid, potentially by enabling these cells to evade the drug’s cytotoxic effects (Acikgoz et al., 2021; Milone et al., 2013). To reinforce this, Tong et al*.* have shown that the SMO receptor inhibitor GDC-0449 and the GLI transcription factor inhibitor GANT-61 effectively inhibited PCSC proliferation by activating apoptotic pathways through SHH signalling (Tong et al., 2018). Interestingly, cyclopamine, an SMO inhibitor of the Hh signalling pathway, is effective in the suppression of the growth of the AR-dependent and AR-independent PCa cells (Chen M. et al., 2010). Moreover, genistein and sulforaphane, which are natural compounds, have shown promising effects in targeting PCSC markers, including CD44 and CD133, respectively. Treatment with these agents led to a significant reduction in tumor growth, both *in vitro* and *in vivo*, highlighting their potential role as complementary therapies against PCSCs (Zhang et al., 2012; Xuan et al., 2024).

In parallel, miRNAs have emerged as key post-transcriptional regulators of gene transcription that are important in PCSCs regulation. The interplay between the dysregulated signalling pathways and the aberrant expression of miRNAs forms a complex regulatory network that sustains the stemness, survival, and therapy resistance of PCSCs (Medrano-Silva et al., 2025; Mao et al., 2025). Therefore, therapeutic strategies aimed at simultaneously targeting these signalling cascades and restoring normal miRNA expression offer promising potential for eliminating PCSCs and overcoming treatment resistance in metastatic PCa (Ramesh et al., 2023). Figure 2 depicts the key signalling pathways involved in the regulation of PCSCs, including the Wnt/β-catenin, Notch, Hedgehog, and PI3K/AKT/mTOR cascades, which converge to modulate the expression of the critical stemness-associated genes, namely CD44, CD133, OCT4, SOX2, NANOG, and MYC. Together, these pathways make up a regulated network of interactions that maintains the stem-like characteristics of PCSCs, contributing to tumor heterogeneity, metastasis, and therapeutic resistance.

**FIGURE 2:**
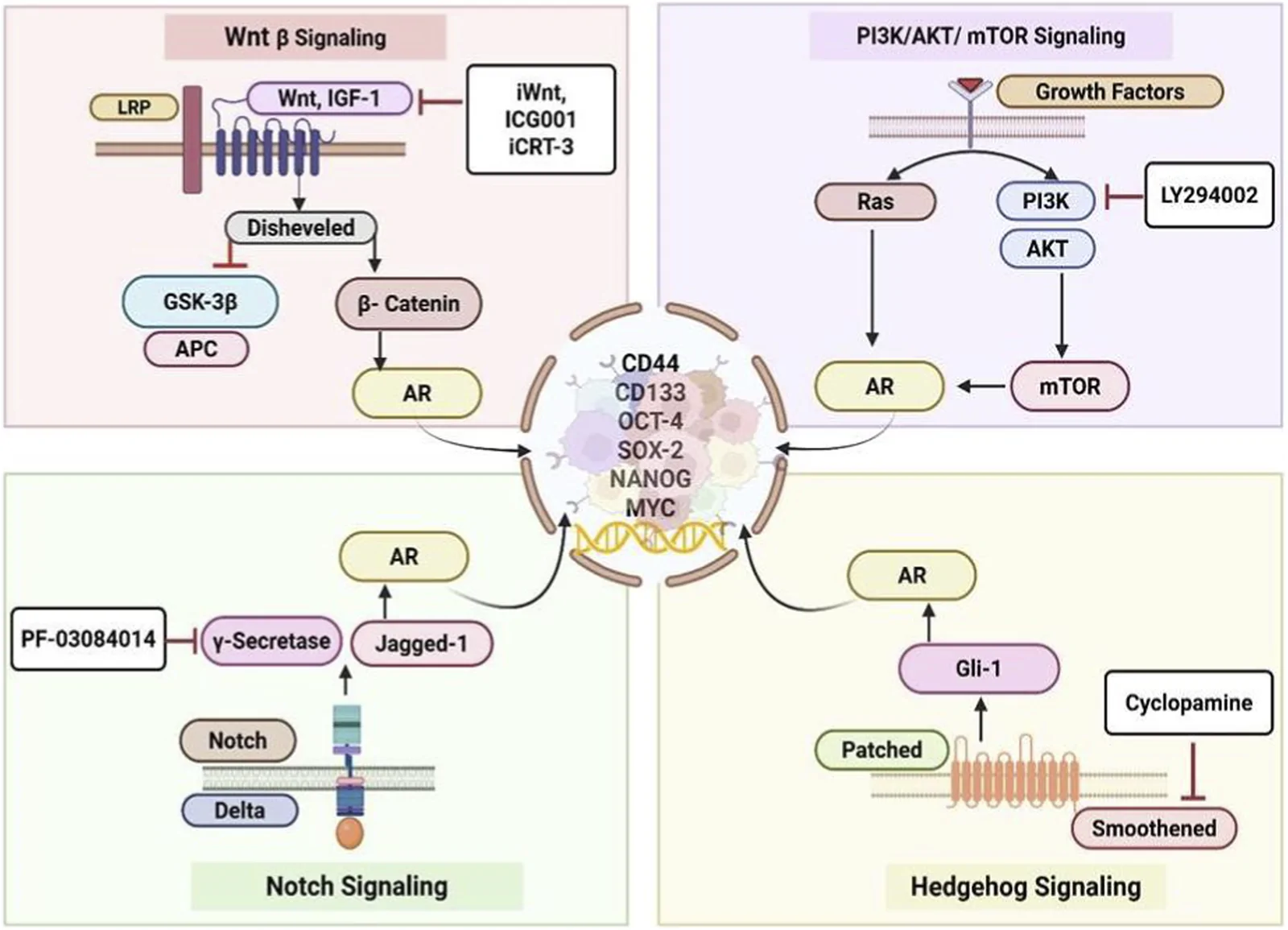
Signalling pathways in regulation of prostate cancer stem cells. PCSCs are maintained through the activation of multiple developmental pathways, including Wnt/β-catenin, PI3K/AKT/mTOR, Notch, and Hedgehog signalling. Aberrant Wnt/β-catenin signalling promotes AR activation, while growth factors for PI3K/AKT/mTOR signalling function downstream and enhance PCSC survival and proliferation. Notch signalling, mediated through Delta-Notch interactions and Jagged-1, contributes to self-renewal, whereas Hedgehog signalling drives stemness and tumorigenicity. These pathways converge to regulate key stemness markers, including CD44, CD133, OCT-4, SOX-2, NANOG, and c-MYC, critical for therapy resistance and disease progression. Pharmacological inhibitors, such as iWnt, ICG001, iCRT-3 (Wnt), LY294002 (PI3K), PF-03084014 (Notch), and cyclopamine (Hedgehog), represent potential strategies to disrupt PCSC signalling and overcome resistance in PCa. This figure was created using BioRender and accessed on 2 September 2025.

### Altered miRNA expression and function in prostate cancer stem cells

miRNAs are ∼22-nucleotide non-coding RNAs that regulate gene expression at the post-transcriptional level. Their biogenesis begins with the processing of primary transcripts by the Drosha-DGCR8 complex in the nucleus into precursor miRNAs, which are exported to the cytoplasm *via* the Exportin-5/Ran-GTP complex. In the cytoplasm, Dicer cleaves the pre-miRNA into a ∼20–25 nucleotide duplex, from which one strand is loaded into the RNA-induced silencing complex (RISC). The seed region (nucleotides 2–8) guides the miRNA to partially complementary sequences in the 3′untranslated regions (3′-UTRs) of target mRNAs, leading to either translational repression or mRNA degradation. Aberrant expression of miRNAs is commonly observed in PCa, highlighting their role in tumor progression and carcinogenesis (de Rooij et al., 2022; Hynes and Kakumani, 2024; Lee and Shin, 2017; O’Brien et al., 2018). Figure 3 represents the biogenesis and regulatory pathways of miRNAs, emphasizing their essential role in gene expression control.

**FIGURE 3:**
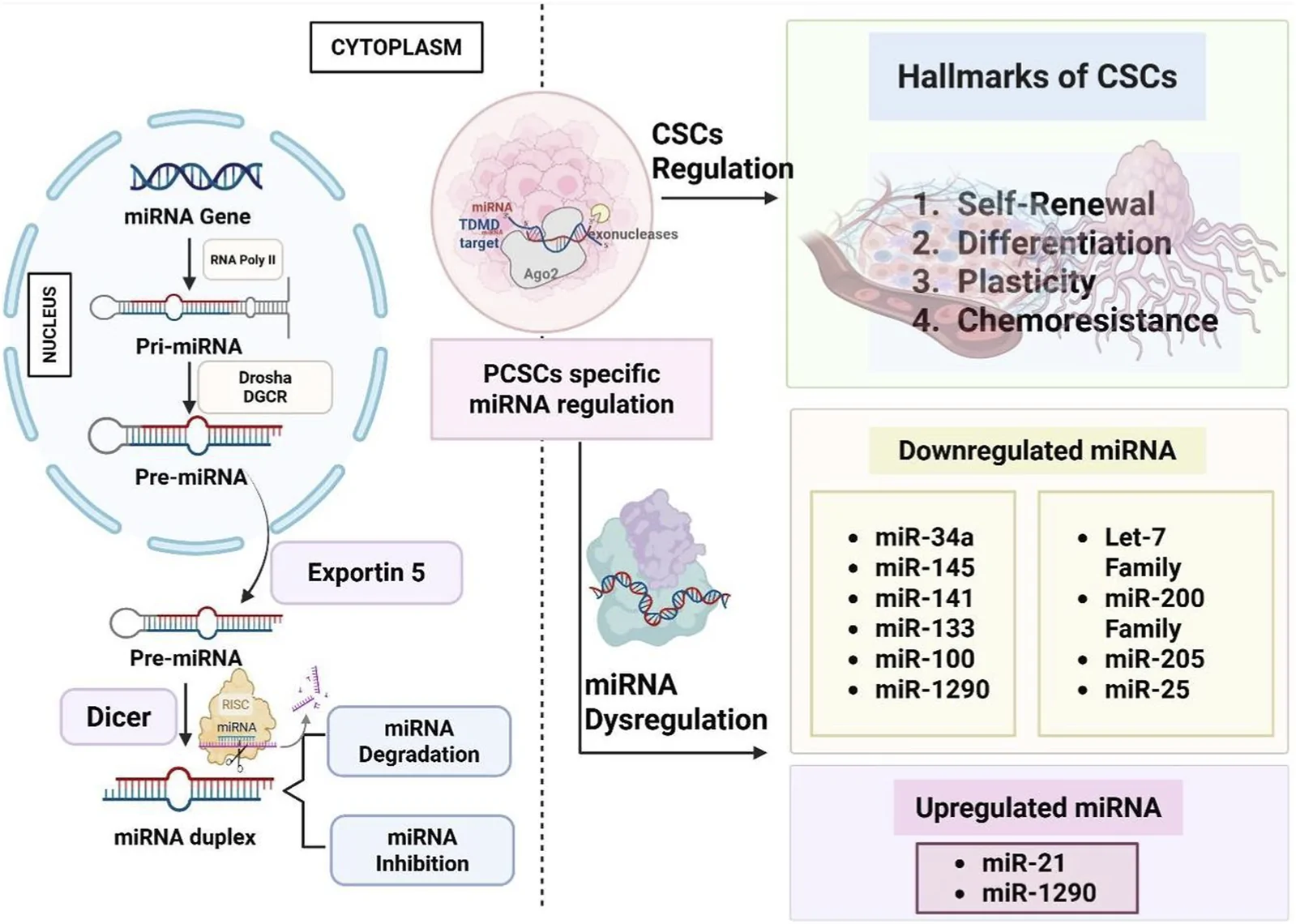
miRNA biogenesis, cancer stem cells regulation, and miRNA dysregulation. Schematic representation of the biogenesis of miRNAs from transcription to maturation and their regulatory functions in PCSCs. Primary-miRNAs are transcribed by RNA polymerase II and processed by the Drosha-DGCR8 complex into pre-miRNAs, which are exported to the cytoplasm *via* Exportin-5. Further cleavage by Dicer generates mature miRNA duplexes, which are incorporated into the RISC complex to mediate mRNA degradation or translational inhibition. Dysregulation of PCSC-specific miRNAs influences hallmark features of CSCs, including self-renewal, differentiation, plasticity, and chemoresistance. Several tumor-suppressive miRNAs, such as miR-34a, miR-145, miR-141, miR-133, miR-100, miR-1290, Let-7 family, miR-200 family, miR-205, and miR-25, were frequently downregulated, whereas oncogenic miR-21 is commonly upregulated, collectively driving CSC-associated malignant progression and therapeutic resistance. This figure was created using BioRender and accessed on 11 September 2025.

## Molecular mechanism of dysregulated miRNA

The molecular mechanisms of dysregulated miRNAs contribute to PCa pathogenesis primarily involve post-transcriptional regulation of gene expression, influencing key cellular processes such as proliferation, apoptosis, EMT, metastasis, and therapy resistance. In PCa, aberrantly expressed miRNAs can act as either oncogenic miRNAs (oncomiRs) or tumor-suppressive miRNAs, depending on the function of their target genes (Kocic, 2024; Yuan et al., 2024).

Tumor-suppressive miRNAs - such as miR-34a, the miR-200 family, miR-145, let-7, miR-1290, miR-100, miR-205, miR-133a-3p, miR-141, and miR-25, frequently downregulated in PCa. Their loss leads to the upregulation of oncogenes and stemness-associated transcription factors, including SOX2, OCT4, and NANOG, as well as activation of signalling pathways such as Wnt, Notch, and EMT, which collectively promote CSC maintenance, tumor invasiveness, and metastatic progression. Conversely, oncogenic miRNAs, such as miR-21, are commonly upregulated in PCa and promote tumor progression by targeting tumor suppressors like PTEN, PDCD4, and MMPs. The dysregulation activates the key signalling pathways, notably the PI3K/AKT signalling cascade, thereby enhancing cell survival, stemness properties, and metastatic potential (Mekhail et al., 2014; Javed et al., 2021; Bilal et al., 2022; de Almeida et al., 2019; Gujrati et al., 2023).

### MicroRNA-34a

Among the best-characterized tumor-suppressive miRNAs, miR-34a plays a critical role in repressing PCSCs. Although TCGA data show increased miR-34a (hsa-miR-34a-5p) expression in primary PCa tissues compared to normal controls (Li W.(Jess) et al., 2022). Functional studies reveal its consistent downregulation in PCSC-enriched populations, such as CD44^+^, CD133^+^, and α2β1^+^ cells (Liu et al., 2011; Liu C. et al., 2012). miR-34a suppresses tumorigenicity by directly targeting CD44, thereby impairing prostasphere formation, cell migration, invasion, and tumor initiation (Yan et al., n.d.). Its transcription is positively regulated by the tumor suppressor p53, which also inhibits CD44 through non-canonical promoter binding, reinforcing a dual regulatory p53-miR-34a-CD44 axis in prostate tumor suppression (Godar et al., 2008). Functionally, miR-34a also targets pathways associated with therapy resistance, including Notch and Wnt signalling. By repressing JAG1, Notch1, and WNT1, miR-34a interferes with β-catenin translocation and TCF7-mediated transcription, curbing metastatic potential (Chen et al., 2014; Dong et al., 2020).

### Let-7 family

Among the earliest identified and most evolutionarily conserved tumor-suppressive miRNA families, the let-7 family plays a pivotal role in regulating cellular differentiation, proliferation, and self-renewal processes (Ma et al., 2021; Nadiminty et al., 2012a). A key post-transcriptional regulator of this family is the RNA-binding protein Lin28, which strongly inhibits the biogenesis and maturation of let-7c, thereby limiting its functional activity (Iliopoulos et al., 2009). In the context of PCa, let-7c is significantly downregulated in clinical specimens and shows an inverse correlation with AR expression, whereas Lin28 expression is positively associated with AR levels (Medrano-Silva et al., 2025; Khan et al., 2019). Lin28 is frequently overexpressed in aggressive PCSCs, where it blocks let-7 processing and prevents the miRNA from targeting oncogenes and stemness regulators such as HMGA2, LIN28, MYC, RAS, and SOX2 - key effectors essential for sustaining self-renewal and pluripotency (Cotino-Nájera et al., 2024; Nadiminty et al., 2012b; Degrauwe et al., 2016). Liu et al*.* evaluated that downregulation of let-7 promotes the expansion of PCSC progenitor populations, including CD44^+^, CD133^+^, integrin α2β1^+^ cells, and the side population, all hallmarks of therapy resistance and stem-like behavior in PCa (Liu C. et al., 2012). Albino et al*.*, demonstrated that the transcription factor ESE3/EHF, expressed in normal prostate epithelial cells, drives differentiation by activating epithelial lineage genes and repressing self-renewal regulators such as Nanog, OCT4, BMI-1, and EZH2, thereby facilitating acquisition of PCSC traits (Albino et al., 2016). Complementing these findings, Jessica et al*.*, revealed that Lin28B promotes therapy-induced neuroendocrine prostate cancer (t-NEPC) by repressing let-7, leading to de-repression of HMGA2, which in turn activates SOX2 transcription, establishing a neuroendocrine and stem-like transcriptional program (Lovnicki et al., 2020). Kong et al*.* reported that overexpression of platelet-derived growth factor-D in PC3 cells induces EMT and elevates expression of stemness markers, including NOTCH1, SOX2, NANOG, OCT4, and Lin28B, accompanied by increased clonogenicity, prostasphere formation, and *in vivo* tumorigenicity (Kong et al., 2010; Kasper, 2009). Therapeutically, berbamine, when combined with cabazitaxel, has been shown to enhance anti-PCSC efficacy by upregulating let-7 expression and downregulating targets such as IGF2BP1 and phospho-STAT3, thereby amplifying the cytotoxic response (Wang et al., 2024).

### MicroRNA-145

miR-145 is another well-established tumor-suppressive miRNA that plays a crucial role in regulating CSC properties such as self-renewal, differentiation, EMT, and overall tumor progression in PCa (Coradduzza et al., 2022). Despite robust evidence supporting its anti-tumor functions, miR-145 is inversely correlated with AR levels, tumor aggressiveness, metastatic potential, resistance to ADT, and poorer patient survival outcomes (Larne et al., 2015; Chen X. et al., 2010). Dong et al*.* revealed that in primary PCa specimens, ZEB2 expression negatively correlates with miR-145 levels, and its upregulation is positively associated with bone metastasis, elevated PSA levels, and higher Gleason scores, underscoring its contribution to PCa aggressiveness (Ren et al., 2014). As a direct transcriptional target of p53, miR-145 has been shown to suppress bone metastasis in PCa by modulating both EMT and CSC-associated pathways (Ren et al., 2013). Functionally, miR-145 directly targets several core stemness regulators, including OCT4, SOX2, and KLF4, thereby impairing the maintenance of pluripotency in both PCa cells and human embryonic stem cells (Xu et al., 2009; Ren et al., 2014). In line with this, Huang et al*.* demonstrated that miR-145 significantly inhibited PC-3 cell viability, colony formation, and tumorsphere generation in PCa cells derived from bone metastases. Moreover, miR-145 is downregulated in key CSC and stemness markers, including CD133, CD44, OCT4, c-MYC, and KLF4 (Huang et al., 2012). Ozen et al*.*, reported that ectopic expression of miR-145-5p in PCa cells led to a significant reduction in SOX2 expression, emphasizing its role in regulating stemness and prostate tumorigenesis (Ozen et al., 2015). Maryam et al*.*, showed that combining paclitaxel with miR-145 resulted in enhanced anti-tumor efficacy by significantly inhibiting PC3 cell proliferation, migration, and stem-like features. This combinatorial approach downregulated several genes linked to metastasis and drug resistance, including MMP-2, MMP-9, CD44, SOX2, and MDR1 (Tohidast et al., 2023). Liu et al*.*, demonstrated that curcumin treatment of CD44^+^/CD133^+^ PCa stem-like cells suppresses stemness by upregulating miR-145 and downregulating lncRNA-ROR, a long non-coding RNA that acts as a competitive endogenous RNA for miR-145. This shift enhances miR-145, leading to OCT-4 repression and reduced expression of genes involved in cell cycle and self-renewal, ultimately inhibiting PCSC tumorigenicity and proliferation (Liu T. et al., 2017).

### MicroRNA-200 family

The miR-200 family, which includes miR-200a, miR-200b, miR-200c, miR-141, and miR-429, acts as a potent group of tumor-suppressive miRNAs in PCa. These miRNAs play a critical role in regulating EMT, maintaining cell polarity, suppressing cancer stemness, and reducing the metastatic potential of PCSCs and advanced-stage tumors (Cavallari et al., 2021; Liu Y.- et al., 2012). Downregulation of miR-200 family members, particularly miR-200a and miR-141, correlates with increased AR signalling and tumor aggressiveness (Cavallari et al., 2021; Waltering et al., 2011; Williams et al., 2013; Xiao et al., 2012). In particular, Guan’s study highlighted the pivotal role of miR-200a in modulating AR signalling during PCa progression. Importantly, BRD4 was identified as a direct target of miR-200a and a key mediator of its regulatory function in oncogenesis (Guan H. et al., 2019). Moreover, miR-200a is known to suppress EMT by targeting ZEB1 and ZEB2, and also modulates stem-like traits by downregulating pluripotency-associated transcription factors such as Bmi1, NANOG, OCT4, and SOX2 in ARCaP and PC3 PDGF-D PCa cells (Kong et al., 2010; Kong et al., 2009). Liu et al. (2017a), emphasized the tumor-suppressive role of miR-141, showing its significant downregulation in stem/progenitor-like PCa populations in both xenograft and primary patient tumors. Restoration of miR-141 expression inhibited key cancer stem cell-like features, including holoclone and sphere formation, invasion, tumor regeneration, and metastasis. Mechanistically, miR-141 targets regulators of cytoskeletal organization and stemness such as CDC42, CDC42EP3, RAC1, ARPC5, CD44, and EZH2 (Xu et al., 2009). In line with these findings, Nanta et al*.*, demonstrated that NVP-LDE-225 (Erismodegib), a Smoothened inhibitor, upregulates the miR-200 family, contributing to inhibition of proliferation, EMT, apoptosis, and PCa stemness. The anti-cancer effects of NVP-LDE-225 are mediated through suppression of the Hedgehog signalling pathway, marked by downregulation of Gli1 and Gli2, as well as modulation of Bcl-2 family proteins, inhibitor of apoptosis proteins, cyclin D1, c-MYC, and Bmi-1. These results underscore the therapeutic potential of targeting the Hedgehog-miR-200 regulatory axis in advanced and stem-like PCa phenotypes (Nanta et al., 2013).

### MicroRNA-133a

miR-133a plays a crucial role in PCa regulation, functioning as a tumor suppressor gene and inversely correlated with patient survival. In PCa tissue and cell lines, both miR-133a-3p and miR-133a-5p are downregulated (Tang et al., 2018). According to the TCGA database, Zheng et al*.*, showed that there is downregulation of miR-133a-5p, leading to activation of AR signalling pathway genes such as AR, FUS, EGFR, and IGFR, underscoring its role in AR signalling modulation (Zheng et al., 2020; Zheng et al., 2020). Furthermore, the reduced miR-133a-3p expression is positively correlated with adverse clinicopathological characteristics, including elevated serum PSA levels, Gleason score, and advanced TNM stages (Kumar et al., 2024). Tang et al*.*, reported that miR-133a-3p targets multiple cytokine receptors of Akt signalling and leads to activation of EGFR, FGFR1, IGF1R, and MET. This aberrant activation promotes increased proliferation, recurrence, and distant metastasis, as well as enhanced PCa bone metastasis (Tang et al., 2018). Hence, miR-133a-3p is associated with increased CSCs regulation and pluripotency-associated markers. Elevated miR-133a-3p leads to reduced spheroid formation and suppression of key stemness markers, including NANOG, BMI-1, OCT4, and SOX2 (Tang et al., 2018). Collectively, these findings underscore the role of miR-133a in PCa aggressiveness, metastasis, and treatment resistance.

### MicroRNA-100

miR-100 plays a crucial role in PCa tumorigenesis, functioning as a tumor suppressor gene; however, Ye et al*.*, emphasized that there was increased proliferation, migration, and invasion by targeting the mTOR gene (Wang et al., 2014). The growing evidence shows that miR-100 targets CDKN1A, CDK6, RB1, ATM, CASP3, EGF, and EFG genes, thereby contributing to enhanced progression, CRPC, and therapeutic resistance (Rane et al., 2016). Altogether, Katia et al*.*, revealed that there is an increased Gleason score in PCa patients with low expression of miR-100 and identified THAP2, SMARCA5, and BAZ2A as its downstream targets (Leite et al., 2013). Wang et al*.*, reported that in primary PCa, there is a negative correlation between miR-100 and Gleason score, as well as total and free PSA levels, which influences the CSCs marker genes while upregulating CD44, c-MYC, OCT4, and KLF4 (Wang et al., 2014). Taken together, these findings underscore the role of miR-100 as a promising therapeutic target for mitigating PCSCs proliferation, migration, and invasion.

### MicroRNA-205

miR-205 is a well-characterized tumor suppressive miRNA in PCa, plays a crucial role in cancer proliferation, migration, invasion, and distant metastasis. In normal prostate epithelium, it is abundantly expressed in the basal cell layer, maintaining cellular homeostasis (Chai et al., 2025; Chauhan et al., 2022). The marked downregulation of miR-205 leads to increased Gleason scores, EMT, and metastatic lesions, and predicts disease progression (Wang N. et al., 2013). Boll et al*.*, revealed that miR-205 is regulated by AR signalling, and its downstream modulation of the MAPK pathway contributes to the progression towards CRPC (Boll et al., 2013). The plausible evidence by Gandellini et al*.*, suggests that miR-205 promotes mesenchymal-to-epithelial transition by upregulation of E-cadherin and downregulation of oncogenes such as interleukin 6, EZH2, N-chimaerin, ErbB3, E2F1, E2F5, ZEB2, and protein kinase Cε (Gandellini et al., 2009). Interestingly, Gandellini et al*.*, in another study, showed the association between miR-205 with cancer-associated fibroblast, regulates stem-like traits in PCa by increasing prostasphere formation and presence of CD44high/CD24low and CD133 positive subpopulations (Gandellini et al., 2014). Jiang et al*.*, from the microarray data, have proved that miR-205 targets RHPN2, a rhophilin Rho GTPase binding protein 2. They showed that exosomal miR-205, which was derived from human bone marrow mesenchymal stem cells, significantly reduces PCa proliferation, migration, and invasion *in vitro*. This effect is mediated by suppression of proliferation markers such as Ki67 and PCNA, MMP-2 and MMP-9 invasion markers, and Bcl2 and Bax apoptosis-related proteins (Jiang et al., 2019). In concordance, Martin et al*.*, evaluated that miR-205 is markedly downregulated in docetaxel-resistant PCSCs, contributing to the increase in stemness markers such as CD44 and CD133, elevation in invasion properties like E-cadherin and vimentin, and loss of epithelial characteristics (Puhr et al., 2012). Together, these findings show that miR-205 acts as a key modulator in PCa proliferation, migration, and invasion, and its dysregulation contributes to CRPC and drug resistance.

### MicroRNA-25

miR-25, another well-known tumor suppressive miRNA, is downregulated in PCa. In multiple studies, it was evidenced that miR-25 downregulation leads to poor patient survival, increased recurrence, and mortality (Puhr et al., 2012). Although miR-25 is normally expressed in the luminal compartment, its activity is markedly reduced in PCa tissues and cell lines, accompanied by an increased expression of stem cell-associated markers such as α2β1high/CD133^+^ (Zoni et al. 2015). In line with these findings, Zoni et al*.*, uncovered a dynamic and differentiation-associated expression pattern of miR-25 in human PCa. In both PCa cell lines and patient-derived primary prostate tumors, miR-25 expression was markedly low in the α2β1hi/CD133+ CSC subpopulation, which is enriched for self-renewal and tumor-initiating potential. Its expression increased progressively during cellular differentiation, first appearing in α2β1hi/CD133-transit-amplifying cells, and subsequently becoming prominent in α2β1low committed basal cells destined for terminal differentiation (Zoni et al., 2015). Overall, these findings concluded that dysregulation of miR-25 plays a significant role in PCa pathogenesis, CSCs regulation and differentiation, and ultimately contributes to treatment failure.

### MicroRNA-1290

miR-1290 plays a crucial role in PCa tumorigenesis; it functions as an oncogenic as well as a tumor suppressor. Multiple studies reported that miR-1290 is associated with poor patient survival, metastasis, invasion, and resistance to therapy (Ghafouri-Fard et al., 2021; Guz et al., 2022). Huo et al*.*, identified miR-1290 as a critical regulatory node implicated in enzalutamide (Enz) resistance and PCSC progression in CRPC. It was demonstrated that miR-1290 expression is significantly downregulated upon Enz treatment, primarily due to disruptions in the AR/CDR1/circCDR1-AS signalling axis. This downregulation leads to the upregulation of BMP4, a key factor that enhances stemness features and therapeutic resistance in PCa. Notably, circCDR1-AS was shown to markedly increase sphere-forming efficiency and elevate the expression of core stemness markers such as CD133 and BMP4 in PCa cells (Huo et al., 2025). Li et al*.*, revealed the role of miR-1290 as an oncogene by targeting its gene RAR Related Orphan Receptor A (RORA) in PCa tumorigenesis, both *in vitro* and *in vivo* (Li Y. et al., 2022). Wang et al*.*, had performed RNA sequencing analysis and showed that miR-1290 functions as an oncogene that promotes metastasis *via* downregulating the GSK3β/β-catenin signalling pathway (Wang S. et al., 2022). Collectively, these findings underscore the pivotal role of miR-1290 in modulating the expression of its target gene and can be used as a promising therapeutic strategy for advanced PCa disease.

### MicroRNA-21

Several oncogenic miRNAs (oncomiRs) have been identified that contribute to PCa initiation, stemness, and progression. The miR-21 is among the most prominently upregulated in the oncomiRs of various stages of PCa. Its expression is significantly higher in localised and metastatic tumors compared to BPH, and significantly correlated with adverse clinical outcome (Seputra et al., 2021a; Seputra et al., 2021b). miR-21 is an AR-responsive miRNA that plays a pivotal role in the maintenance of PCa cell proliferation, survival, and self-renewal. Its expression is progressively upregulated during the transition from androgen-dependent PCa to CRPC, underscoring its role in the disease progression and resistance to therapy (Guan C. et al., 2019; Stafford et al., 2022), (An overview of prostate cancer (PCa) diagnosis: Potential role of miRNAs, 2022). Mechanistically, miR-21 exerts its oncogenic activity by silencing key tumor suppressor genes, including PTEN and PDCD4, thereby activating the PI3K/AKT/mTOR signalling cascade, which is a core pathway in cancer cell growth, survival, and stemness (Chawra et al., 2024). Shukla et al*.*, evaluated that miR-21 promotes the transformation of prostate tumor cells into a stem-like phenotype characterized by CD44+/CD24-and CD44+/CD133+ surface marker expression (Shukla et al., 2023). In addition to this, there is increasingly growing evidence that miR-21 increases other stemness-related markers such as ALDH+ +, ABCG2 +, CD133+, and CD24^−^, reinforcing its role in PCSC maintenance and metastatic competence. miR-21 stimulates the expression of MMP-2, MMP-9, and MMP-13 in fibroblasts, promoting their activation and enhancing the migratory potential of PCa cells, characteristic features of a pro-tumorigenic microenvironment (Sánchez et al., 2015). Bao et al*.*, reported that hypoxia, commonly found in the tumor microenvironment, drives the prostasphere formation and increases the expression levels of core stemness factors, including NANOG, OCT4, EZH2, CD44, and EpCAM (Bao et al., 2012). Combined, these observations underscore miR-21’s role as an oncogenic driver that bridges androgen signalling, PCSC expansion, microenvironmental modulation, and therapeutic resistance in metastatic PCa.

The right side of Figure 3 depicts that miRNA regulation influences PCSCs and miRNA. PCSC-specific miRNAs govern key hallmarks of CSCs such as self-renewal, differentiation, plasticity, and chemoresistance, thereby contributing to tumor growth and treatment resistance. The interplay between a tumor-suppressive and oncogenic miRNA reflects their crucial role in maintaining PCSC biology and emphasizes their potential role as a promising therapeutic target for combating PCa progression and treatment failure.

Table 2 summarizes and highlights the key miRNAs used in PCSC, their major targets, associated pathways, and functional roles.

**TABLE 2:** Key miRNAs regulating prostate cancer stem cells: targets, functional roles, and references.

Micro RNA	Key targets/pathways	Functional role	References
miR-34a	CD44, JAG1, Notch1, WNT1	Inhibits prostasphere formation, invasion, and tumor initiation	Larne et al. (2015), Ren et al. (2014), Ren et al. (2013)
Let-7c	HMGA2, LIN28, MYC, RAS, SOX2	Suppresses self-renewal, differentiation, and therapy resistance	Waltering et al. (2011), Chen et al. (2010b)
miR-145	OCT4, SOX2, KLF4, CD44, c-MYC	Inhibits stemness, EMT, metastasis, and bone colonization	Coradduzza et al. (2022), Chauhan et al. (2022), Chai et al. (2025), Wang et al. (2013b)
miR-200 family (miR-200a, 200b, 200c, 141, 429)	ZEB1, ZEB2, BRD4, Bmi1, SOX2, NANOG, EZH2	Inhibits EMT, invasion, pluripotency, and metastasis	Kong et al. (2009), Xiao et al. (2012), Cavallari et al. (2021)
miR-141	CDC42, RAC1, ARPC5, CD44, EZH2	Suppresses holoclone/sphere formation and metastasis	Xiao et al. (2012)
miR-133a-3p	EGFR, FGFR1, IGF1R, MET	Suppresses PI3K/AKT, pluripotency markers	Tang et al. (2018), Kumar et al. (2024)
miR-100	c-MYC, OCT4, KLF4	Inhibits EMT, invasion, and stemness	Wang et al. (2014), Rane et al. (2005a)
miR-205	EMT/stemness regulators, possibly ZEB1, EZH2	Maintains epithelial traits, blocks stemness	Chai et al. (2025), Chauhan et al. (2022), Boll et al. (2013), Gandellini et al. (2014)
miR-25	—	Expressed during the differentiation of PCSCs	Zoni et al. (2015)
miR-1290	BMP4 (de-repressed due to low miR-1290)	Regulates Enz resistance, promotes stemness *via* BMP4	Huo et al. (2025)
miR-21	PTEN, PDCD4, promotes MMPs, activates PI3K/AKT	OncomiR: Promotes proliferation, stemness, therapy resistance	Guan et al. (2019a), Stafford et al. (2022), Chawra et al. (2024), Shukla et al. (2023)

Overall, targeting PCSCs with miRNA-based interventions, augmented by CRISPR/Cas9, nanomedicine, CAR-T cell-based therapies, and epigenetic reprogramming, represents a promising approach to overcome therapy resistance and enhance clinical outcomes in advanced PCa.

## Therapeutic applications of microRNA-mediated cancer stem cell modulations

### CRISPR/Cas-9 mediated modulation of CSCs and microRNA

CRISPR/Cas9 is a revolutionary therapeutic platform, as it can edit genes precisely and efficiently edit disease-associated genes (Guo et al., 2022). Unlike traditional therapies, which often focus on downstream signalling, CRISPR enables direct manipulations of genetic drivers of disease, thereby offering the potential for permanent correction of pathogenic mutations (Ramesh et al., 2023). Its versatility extends to targeting oncogenes, tumor suppressors, and regulatory non-coding RNAs such as miRNAs, positioning it as a valuable tool in the management of complex malignancies, including PCa (Macarrón Palacios et al., 2024; Ravichandran and Maddalo, 2023; Garg et al., 2025). CRISPR/Cas9 can be engineered to disrupt pathways that maintain CSCs, thereby overcoming therapy resistance and preventing metastatic progression (Chehelgerdi et al., 2024). For instance, Bhat et al*.*, showed that the CRISPR/Cas9-mediated knockdown of GD3 synthase inhibited GD2 production and suppressed CSC-like characteristics in PCa (Bhat et al., 2024). Similarly, Lovnicki et al*.*, have found that treatment-induced neuroendocrine PCa promotes the stem-like reprogramming and neuroendocrine differentiation that depends on activation of the LIN28B/let-7/SOX2 axis, but depletion of LIN28B by CRISPR/Cas9 inhibitors significantly decreases tumor growth (Lovnicki et al., 2020). Wet et al., identified SOX2 as an essential transcription factor in PCa, driving metastatic progression, lineage plasticity, and therapeutic resistance, and highlighted that SOX2 knockdown with CRISPR/Cas9 can be a promising therapeutic strategy (Lotfi et al., 2023). In line with these findings, the integration of CRISPR/Cas9 with nanocarrier-based platforms improves tumor selectivity, reducing off-target action and therapeutic efficacy (Bhattacharjee et al., 2022). Beyond preclinical models, CRISPR/Cas9 has also entered early clinical evaluation in PCa through immune checkpoint modulation strategies, primarily *via ex vivo* PD-1 knockout in autologous T cells. A Phase 1/2 clinical trial (NCT03525652) is currently assessing a therapeutic vaccine in combination with PD-1 knockout T cells for advanced PCa [Clinical trial: Prostate Cancer, (NCT03525652)]. Despite encouraging preclinical evidence for CRISPR-mediated targeting of CSC-related genes and miRNAs in PCa, direct *in vivo* or intratumoral CRISPR/Cas9 gene editing - particularly of CSC-linked miRNAs - has not yet progressed beyond the preclinical stage. Altogether, these studies highlight the potential role of CRISPR/Cas9 and deciphering its functional role in CSC-linked miRNAs in PCa, and can be a promising precision medicine approach to overcome resistance and improve clinical outcomes.

### Nanotechnology mediated modulation of CSCs and microRNA

In recent years, the rapid advancement of nanotechnology has attracted considerable attention from researchers and clinicians for its potential applications in cancer therapy (Su et al., 2024). Unlike conventional therapeutics, nanomedicine offers improved pharmacokinetics, precise drug delivery, and controlled release, while minimizing off-target effects, thereby enhancing safety and allowing for a wider therapeutic dosing range (Li et al., 2023; Gener et al., 2020; Malik et al., 2023). Various engineered nanocarriers, including liposomes, polymeric nanoparticles, dendrimers, gold nanoparticles, and mesoporous silica nanoparticles, have been developed to deliver chemotherapeutics, small molecules, and nucleic acid-based therapies with high specificity (Sultana et al., 2022; Sun et al., 2023). In particular, the delivery of CSC-specific miRNAs has emerged as a promising strategy, given their key role as post-transcriptional regulators of genes involved in CSC maintenance, EMT, and therapy resistance in PCa (Su et al., 2024). For instance, Yang et al*.*, demonstrated that combination therapy using paclitaxel- and cyclopamine-based polymer-drug conjugate micelles effectively overcomes paclitaxel resistance in PCa by targeting CSCs, inhibiting Hedgehog signalling, and restoring tumor-suppressive miRNAs such as miR-29b, miR-34a, miR-148a, and miR-200c (Yang et al., 2017). Mahira et al*.*, reported that hyaluronic acid-coated cationic liposomes co-loaded with cabazitaxel and silibinin efficiently targeted both PCa cells and CSCs by exploiting CD44 receptor overexpression (Mahira et al., 2019). Prakash et al*.*, evaluated the potential role of nanotechnology for diagnostics, showing that DNA-linked gold nanoprobe-based quantification of dysregulated serum miRNAs, including miR-21, miR-141, and miR-375, offers a non-invasive tool for PCa detection (Mahira et al., 2019). Collectively, these studies underscore that integrating miRNA therapeutics with nanotechnology not only provides a novel approach to eliminate CSCs but also enhances diagnostic accuracy, overcomes therapy resistance, and advances precision medicine strategies in PCa.

### CAR-T cell (immunotherapy) mediated modulation of CSCs and microRNA

Chimeric antigen receptor T (CAR-T) cell therapy has emerged as a transformative immunotherapeutic approach, and it has significant potential against solid tumors, such as PCa (Kheyrolahzadeh et al., 2023; Ku et al., 2024). Unlike conventional T cell-based therapies, CAR-T cells are genetically engineered to recognize tumor-associated antigens independently of the major histocompatibility complex, enabling a more robust and sustained anti-tumor response (Lin et al., 2024; Ullah et al., 2018). Some tumor- and CSC-associated antigens, such as prostate-specific membrane antigen (PSMA), prostate stem cell antigen (PSCA), epithelial cell adhesion molecule (EpCAM), CD44, and B7-H3 (CD276), have been explored as possible CAR-T targets and are highly expressed on malignant PCa cells and PCSCs (Zhang et al., 2021; Hadiloo et al., 2025; Srour and Akin, 2023; Zarrabi et al., 2023; Frieling et al., 2023). Supporting this rationale, Wang et al*.*, reported that the dendritic cell-cytokine sensitized killer cell preparations sensitized to immunogenic peptides had potent cytotoxicity activity on PCSCs *in vitro* and *in vivo* through targeting the CSC-associated markers of CD44 and EpCAM, thus suppressing the growth of tumors in PCa xenograft models (Wang Z. et al., 2020). Zhang et al*.*, showed that fractionated irradiation selectively enhances B7-H3 expression on PCSCs that markedly increases the susceptibility to B7-H3 CAR-T cell-mediated cytotoxicity (Zhang et al., 2025). Taken together, these results highlight the therapeutic value of the integration of CAR-T cell immunotherapy with other interventions that modulate CSC biology. Specifically, miRNA-based therapies, capable of inhibiting CSC stemness, EMT, and immunomodulatory responses, provide a compelling complementary approach. Therefore, miRNA modulation combined with CAR-T therapy represents a novel and synergistic strategy to eradicate radioresistant and chemo-resistant PCSCs, reduce tumor recurrence, and ultimately improve outcomes in advanced and metastatic PCa.

### Epigenetic therapy-mediated modulation of CSCs and microRNA

Epigenetic therapy has emerged as a promising strategy in PCa management, as it specifically targets the intrinsic plasticity of CSCs, which are major drivers of therapy resistance, disease recurrence, and metastatic progression (Qin et al., 2020; Wainwright and Scaffidi, 2017; Macedo-Silva et al., 2021). Aberrant epigenetic alterations - including DNA methylation, histone modifications, and dysregulated miRNAs- play pivotal role in maintaining CSC stemness and plasticity, while also facilitating EMT in PCa (Suzuki et al., 2013; Huang et al., 2025). Preclinical studies demonstrate that DNA methyltransferase inhibitors (e.g., 5-aza-2′-deoxycytidine/decitabine) can restore the expression of tumor-suppressive miRNAs such as miR-34a, miR-141, and miR-199a-3p, thereby reducing stemness, clonogenicity, and metastatic potential of PCSCs (Li W.(Jess) et al., 2022). Moreover, combined epigenetic therapy with DNA methyltransferase inhibitors and histone deacetylase inhibitors, such as trichostatin A or vorinostat, has shown synergistic effects by reversing aberrant epigenetic silencing, thereby inducing apoptosis and cell cycle arrest (Suraweera et al., 2018; Tahghighi et al., 2025). Majid et al*.*, demonstrated that miR-34b functions as a tumor suppressor in PCa, where its silencing by CpG hypermethylation is reversed upon re-expression, leading to inhibition of DNMTs and HDACs, suppression of AKT signalling, induction of apoptosis and G0/G1 cell cycle arrest, and suppression of EMT (Majid et al., 2013). Importantly, clinical translation of epigenetic therapy is under active investigation, with epigenetic drugs being tested in combination with AR inhibitors, CSC-targeted approaches, and immunotherapies to enhance treatment efficacy in CRPC. Collectively, by reinstating tumor-suppressive miRNAs and disrupting the epigenetic circuits sustaining CSCs, epigenetic therapy represents a novel therapeutic paradigm with the potential to overcome resistance and improve outcomes in aggressive, treatment-refractory PCa.

Table 3 summarizes the role of the therapeutic approach, key findings, outcome, and conclusion.

**TABLE 3:** Emerging therapeutic strategies targeting prostate cancer stem cells.

Therapeutic approach	Key findings	Outcomes	Conclusion	References
CRISPR/Cas9	CRISPR/Cas9-mediated knockdown of GD3 synthase inhibited GD2 biosynthesis, suppressing CSC-like properties in PCa.	Reduced stemness and tumorigenicity in PCa models.	CRISPR’s ability to target CSC-related genes and reverse therapy resistance.	Bhat et al. (2024)
CRISPR/Cas9	Activation of the LIN28B/let-7/SOX2 axis promotes stem-like reprogramming and neuroendocrine differentiation; CRISPR-mediated depletion of LIN28B suppresses this effect.	Significant reduction in tumor growth and stemness markers.	CRISPR targeting of reprogramming pathways can mitigate neuroendocrine differentiation in advanced PCa.	Lovnicki et al. (2020)
CRISPR/Cas9	Identified SOX2 as a master regulator of lineage plasticity and therapy resistance. CRISPR-mediated SOX2 knockdown inhibited metastasis.	Attenuation of metastatic and therapy-resistant phenotypes.	SOX2 silencing *via* CRISPR/Cas9 is a promising precision strategy to target PCSCs.	Lotfi et al. (2023)
CRISPR/Cas9 with nanocarriers	Integration of CRISPR with nanocarrier-based delivery enhances tumor specificity and reduces off-target effects.	Improved therapeutic efficacy and safety.	Nanocarrier-assisted CRISPR systems provide an advanced platform for CSC-targeted precision medicine.	Lotfi et al. (2023)
Nanotechnology	A combination of paclitaxel and cyclopamine-based polymer-drug micelles targeted Hedgehog signalling and restored tumor-suppressive miRNAs (miR-29b, miR-34a, miR-148a, miR-200c).	Overcame paclitaxel resistance and inhibited CSC proliferation.	Nanocarrier-mediated co-delivery enhances therapeutic response through miRNA restoration.	Yang et al. (2017)
Nanotechnology	Hyaluronic acid-coated cationic liposomes co-loaded with cabazitaxel and silibinin targeted CD44-overexpressing PCSCs.	Efficient targeting of both bulk tumor cells and CSCs.	Dual-drug nanocarriers provide synergistic therapy and minimize systemic toxicity.	Mahira et al. (2019)
Nanotechnology	DNA-linked gold nanoprobes quantified dysregulated serum miRNAs (miR-21, miR-141, miR-375).	Enabled non-invasive detection of PCa-associated miRNAs.	The diagnostic potential of miRNA-integrated nanoplatforms for PCa.	Mahira et al. (2019)
CAR-T	Dendritic cell–cytokine-induced killer cell preparations targeting CD44 and EpCAM suppressed tumor growth in xenograft models.	Enhanced cytotoxicity against PCSCs *in vitro* and *in vivo*.	CSC marker-directed CAR-T therapy shows promise in eradicating resistant PCa populations.	Wang et al., 2020a
CAR-T	B7-H3 CAR-T cells effectively killed PCSCs, especially after fractionated irradiation increased B7-H3 expression.	Increased susceptibility of CSCs to CAR-T-mediated cytotoxicity.	Combining radiotherapy with CAR-T therapy enhances CSC targeting efficiency.	Zhang et al., 2021
Epigenetic therapy	DNA methyltransferase inhibitors (5-aza-2′-deoxycytidine, decitabine) restored tumor-suppressive miRNAs (miR-34a, miR-141, miR-199a-3p).	Reduced clonogenicity, stemness, and metastasis.	Epigenetic reprogramming reinstates miRNA function to suppress CSC traits.	Li et al. (2022a), Liu et al. (2022), Liu et al. (2016)
Epigenetic therapy	Combined DNMT inhibitors with HDAC inhibitors (trichostatin A, vorinostat) reversed epigenetic silencing and induced apoptosis.	Synergistic induction of apoptosis and growth arrest.	Dual epigenetic modulation enhances therapeutic potency and reverses resistance.	Suraweera et al., (2018), Tahghighi et al. (2025)
Epigenetic therapy	Re-expression of miR-34b suppressed DNMTs, HDACs, and AKT signalling; induced apoptosis and G0/G1 arrest.	Inhibited EMT and tumor progression.	Targeting hypermethylated miRNAs restores tumor-suppressive networks and sensitizes cells to therapy.	Majid et al., 2013

Figure 4 outlines emerging therapeutic strategies for treating PCa, emphasizing innovative approaches targeting CSCs. These include CRISPR-based gene editing, which enables precise correction or silencing of oncogenic mutations; nanomedicine, which facilitates targeted drug delivery and minimizes systemic toxicity; CAR-T cell therapy, designed to enhance immune recognition and elimination of tumor cells; and epigenetic therapy, which modulates aberrant gene expression by reversing DNA methylation and histone modifications. Collectively, these strategies aim to overcome limitations of conventional therapies by addressing molecular and cellular mechanisms underlying CSC-driven tumor initiation, progression, and resistance, offering promising avenues for more effective and personalized PCa treatment.

**FIGURE 4:**
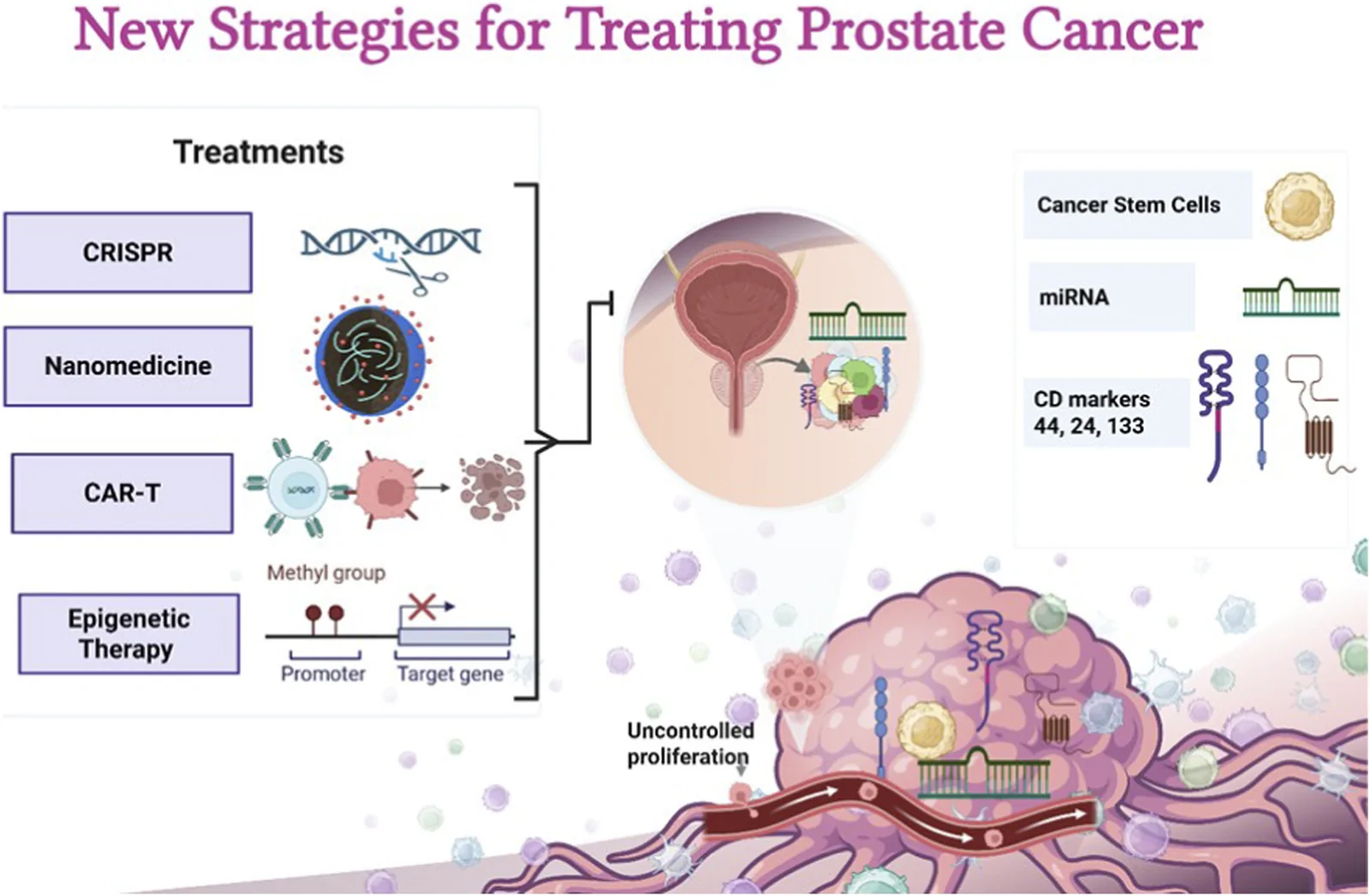
Emerging therapeutic strategies targeting cancer stem cells in prostate cancer. The schematic illustrates novel approaches being developed to counteract CSC-driven tumor progression and therapy resistance. Advanced treatment modalities include CRISPR-based genome editing, Nanomedicine delivery systems, CAR-T cell immunotherapy, and Epigenetic Therapies targeting aberrant gene regulation. These strategies aim to disrupt CSC-associated mechanisms such as miRNA dysregulation and surface marker expression (e.g., CD44, CD24, CD133), which contribute to uncontrolled proliferation, therapeutic resistance, and tumor recurrence. This figure was created using BioRender, and accessed on 30 October 2025.

The schematic illustrates novel approaches being developed to counteract CSC-driven tumor progression and therapy resistance. Advanced treatment modalities include CRISPR-based genome editing, Nanomedicine delivery systems, CAR-T cell immunotherapy, and Epigenetic Therapies targeting aberrant gene regulation. These strategies aim to disrupt CSC-associated mechanisms such as miRNA dysregulation and surface marker expression (e.g., CD44, CD24, CD133), which contribute to uncontrolled proliferation, therapeutic resistance, and tumor recurrence.

## Conclusion and future perspectives

The management of metastatic PCa is a major clinical challenge, primarily due to the persistence of therapy-resistant subpopulations that undermine the long-term efficacy of conventional treatment strategies. Over the past decade, miRNA expression profiles have been widely characterized. However, to date, not many studies have specifically addressed the role of PCSCs-associated miRNAs. In the present review, we have highlighted the pivotal role of CSCs and miRNAs in driving this resistance by regulating self-renewal, differentiation, metastasis, and adaptive signalling pathways. Aberrant miRNA expression orchestrates tumor-repressive or oncogenic networks that maintain CSC phenotypes, enhance metastatic dissemination, and survival under therapeutic pressure. Future experimental investigations are crucial to elucidate the functional roles and regulatory mechanisms of CSC-specific miRNAs and their downstream mRNA targets to elucidate the molecular mechanism of therapeutic resistance in metastatic PCa. Each aberrantly expressed miRNA represents a potential therapeutic target, offering an opportunity to restore normal gene expression. Correcting these dysregulated pathways holds the promise of halting, or even reversing, malignant cellular phenotypes and therapeutic responsiveness. It has been shown that the expression of miRNAs is altered during cancer progression, EMT, and therapy resistance, which may be mitigated through precise modulation of miRNA networks. Translationally, this interplay positions CSC-associated miRNAs as promising biomarkers for disease progression and therapeutic response, while also offering novel molecular targets for intervention. This review emphasizes the significance of PCa-specific miRNAs as promising therapeutic targets due to their involvement in sustaining stem-like phenotypes and enhancing metastatic potential. Rather than focusing solely on eradicating resistant tumor populations, miRNA-based strategies aim to reprogram aberrant regulatory networks and restore pre-malignant cellular states. The integration of advanced platforms such as CRISPR/Cas9-mediated editing, nanomaterial-based delivery systems, CAR-T cell therapy targeting CSC-associated markers, and epigenetic modulators represents a promising approach to overcoming therapeutic resistance. Translating these innovations into clinical practice will be pivotal for achieving durable responses and improving therapeutic outcomes in metastatic PCa.
